# Highly Time-Resolved Apportionment of Carbonaceous Aerosols from Wildfire Using the TC–BC Method: Camp Fire 2018 Case Study

**DOI:** 10.3390/toxics11060497

**Published:** 2023-05-31

**Authors:** Matic Ivančič, Martin Rigler, Bálint Alföldy, Gašper Lavrič, Irena Ježek Brecelj, Asta Gregorič

**Affiliations:** 1 Aerosol d.o.o., SI-1000 Ljubljana, Slovenia; 2 Centre for Atmospheric Research, University of Nova Gorica, SI-5000 Nova Gorica, Slovenia

**Keywords:** black carbon, brown carbon, carbonaceous aerosols, wildfire, secondary organic aerosols

## Abstract

The Camp Fire was one of California’s deadliest and most destructive wildfires, and its widespread smoke threatened human health over a large area in Northern California in November 2018. To analyze the Camp Fire influence on air quality on a 200 km distant site in Berkeley, highly time-resolved total carbon (TC), black carbon (BC), and organic carbon (OC) were measured using the Carbonaceous Aerosol Speciation System (CASS, Aerosol Magee Scientific), comprising two instruments, a Total Carbon Analyzer TCA08 in tandem with an Aethalometer AE33. During the period when the air quality was affected by wildfire smoke, the BC concentrations increased four times above the typical air pollution level presented in Berkeley before and after the event, and the OC increased approximately ten times. High-time-resolution measurements allow us to study the aging of OC and investigate how the characteristics of carbonaceous aerosols evolve over the course of the fire event. A higher fraction of secondary carbonaceous aerosols was observed in the later phase of the fire. At the same time, the amount of light-absorbing organic aerosol (brown carbon) declined with time.

## 1. Introduction

In recent years, the frequency of extreme wildfire events and the total burned area in California have increased due to climate-change-related temperature increases and precipitation decreases [1,2]. Besides the material damage and potential risk to human lives, the wildfires also emit an enormous amount of carbonaceous aerosols (CA), which can be transported to a few hundred kilometers away from the wildfire location, affecting the air quality and public health over a wider area [3,4]. Epidemiological and toxicological studies indicate that CA may be relatively more toxic than other PM_2.5_ compounds, such as soluble salts, due to their high potential to inflict oxidative stress [5,6]. On the other hand, CA influence the Earth’s radiation budget directly through light absorption and scattering or indirectly due to their role in cloud formation [7,8,9].

CA generally combine two chemically different species, black carbon (BC) and highly variable organic aerosols (OA) [10]. BC is an inert fraction that strongly absorbs light. BC is always a consequence of incomplete combustion and is emitted directly from sources as a particle. On the contrary, OA can be primarily emitted as particles (primary OA-POA) or formed in the atmosphere via the oxidation of gaseous precursors (secondary OA-SOA). Brown carbon (BrC) is a fraction of OA that absorbs light. The light absorption by BrC is usually enhanced near the ultraviolet part of the spectra [11,12]. The mass of carbon atoms in CA and OA is called total carbon (TC) and organic carbon (OC), respectively. Similarly, primary OC (POC) and secondary OC (SOC) represent the mass of carbon atoms in POA and SOA. Docherty et al. [13] reported typical POA/POC and SOA/SOC ratios of 1.2 and 1.8, respectively, for the average situation in Los Angeles, California, unaffected by wildfires. During wildfires, the OA/OC ratio usually increases owing to the higher oxidation state of biomass-burning-related (BB) aerosols. For example, Zhou et al. [14] reported OA/OC values between 1.7 and 3.0 when wildfire plumes impacted the air, and similarly, Zhang et al. [7] reported OA/OC ratios between 1.62 and 2.58.

CA emitted from wildfires are mostly organic [7,15]. Like other BB aerosols, fresh wildfire emissions contain a high fraction of BrC [15]. Dark aging can enhance BrC absorption [15], while BrC absorption is decreased with aging during daylight owing to the photobleaching of chromophores [16,17,18].

There are two major pathways of biomass-burning-related SOA formation: SOA formation from either primarily emitted volatile organic compounds (VOCs) or dilution-driven POA evaporation and subsequent SOA formation [19,20]. Amongst the VOCs, the main contributors to SOA formation from wildfires are phenol, benzene, and catechol [21], where gaseous catechol represents the intermediate phase of phenol oxidation to SOA.

Our study reports the extreme pollution of a large wildfire event characterized in detail using high-time-resolution TC and BC measurements. With the TC–BC method, we analyzed the influence of the Camp Fire smoke at a distant site in Berkeley in November 2018. A BC tracer and brown carbon models were used to characterize further the primary and secondary OA and the increased light absorption on BrC. The highly time-resolved measurements also offer to analyze the smoke aging.

## 2. Materials and Methods

### 2.1. Location and Measurements Setup

**Figure 1:**
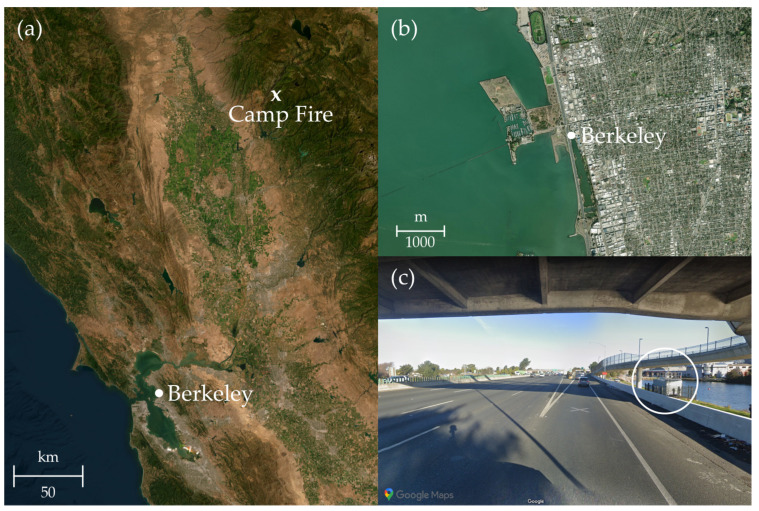
Location of measurement site in Berkeley and the approximate location where Camp Fire started in Northern California, USA (**a**). The site is located near Aquatic Park (**b**), and it is located near the exit of the 10-lane highway (**c**). (**a**,**b**) were plotted with python library basemap using ArcGis WorldImagery, and (**c**) was taken from google maps street view.

The Bay Area Air Quality Measurement District (BAAQMD) site in Berkeley (CA, USA) is located near the Aquatic Park (Figure 1). Its micro-location is near the exit from a 10-lane highway (37.8648, −122.3028), so it is characterized as a traffic station. The Berkeley site is located approximately 200 km away from the Camp Fire center.

In this study, we used the Carbonaceous Aerosol Speciation System (CASS, Aerosol Magee Scientific, Ljubljana, Slovenia), comprising two instruments, a Total Carbon Analyzer, model TCA08 [22], in tandem with an Aethalometer, model AE33 [23]. CASS measured total carbon (TC) and black carbon (BC) with high time resolution. The difference can be inferred as the organic carbon (OC):

OC(t) = TC(t) − BC(t) (1)

TCA08 used a simple thermal protocol: concentrations of TC were measured by the rapid combustion of particles collected on a quartz filter. The sample was heated almost instantaneously to 940 °C, at which the carbonaceous compounds were efficiently combusted to CO_2_. The pulse of CO_2_ created during the combustion phase of the analysis was detected as a large transient increase above the background CO_2_ level. TCA08 had two identical parallel chambers to ensure continuous measurements without dead time. Wherein one channel collected a sample, the other performed analysis of an already collected sample. The limit of detection (LoD) was 0.3 µg/m^3^. TCA was operated at a 30 min resolution, sampling the PM_2.5_ fraction at 16.7 L min^−1^.

The tandem Aethalometer measured the aerosol light absorption and corresponding equivalent BC concentration at seven wavelengths (370, 470, 520, 590, 660, 880, and 950 nm) with LoD equal to 0.03 µg/m^3^. The sample was collected on a glass fiber filter with a flow rate of 5 L min^−1^ and a PM_2.5_ size-selective inlet. Light attenuation was measured at a time resolution of 1 min. The Aethalometer used a ‘dual-spot’ methodology to correct the filter-loading effect in real-time [23]. The absorption coefficient (b_abs_) was calculated from the attenuation coefficient (b_ATN_) using a multiple-scattering parameter C:

(2) $$b_{\mathrm{abs}}\left(\lambda ,t\right)=\frac{b_{\mathrm{ATN}}\left(\lambda ,t\right)}{C}$$

The equivalent BC mass was calculated from b_abs_ using mass absorption cross section (MAC):

(3) $$\mathrm{BC}\left(t\right)=\frac{b_{\mathrm{abs}}\left(880\;\mathrm{nm},t\right)}{\mathrm{MAC}\left(880\;\mathrm{nm}\right)}$$

In Equations (2) and (3), the manufacturer default values were used for C and MAC (880 nm): 1.39 and 7.77 m^2^/g, respectively.

The advantages of using CASS compared to online and offline OC/EC analyzers include high time resolution, no sampling dead time, online filter-loading effect compensation for BC measurements, and low maintenance [23]. As the thermal measurements were performed without a fragile quartz sample oven, high-purity gases, and a catalyst, they are suitable for field campaigns [22].

### 2.2. BC Tracer Model and Brown Carbon Model

**Figure A1:**
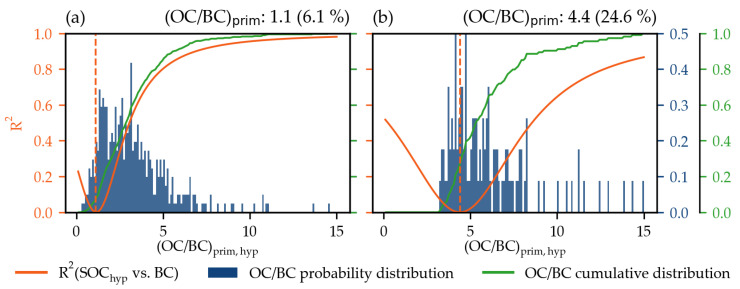
BC tracer model before/after Camp Fire event (**a**) and when the air was affected by Camp Fire smoke (**b**). OC/BC probability distribution is presented with blue and cumulative OC/BC distribution with a green line. The orange curve shows the R^2^ score between the hypothetical SOC and BC for different hypothetical (OC/BC)_prim_ ratios. The vertical dashed orange line marks the minimum of the R^2^ function (optimal value). The optimal value is also written in the title with the calculated percentile in brackets.

To split OC into primary OC (POC) and secondary OC (SOC), we used the BC tracer model [10,24]:

(4) $$\mathrm{POC}\left(t\right)\;=\;{\left(\frac{\mathrm{OC}}{\mathrm{BC}}\right)}_{\mathrm{prim}}\cdot \;\mathrm{BC}\left(t\right)\;\mathrm{SOC}\left(t\right)\;=\;\mathrm{OC}\left(t\right)\;-\;\mathrm{POC}\left(t\right)\;=\;\mathrm{OC}\left(t\right)\;-\;{\left(\frac{\mathrm{OC}}{\mathrm{BC}}\right)}_{\mathrm{prim}}\cdot \;\mathrm{BC}\left(t\right)$$

where (OC/BC)_prim_ is a time-independent ratio and is expected to be source-dependent. To find the optimal (OC/BC)_prim_ ratio, the minimum R-squared (MRS) [25,26] method was used. Briefly, the hypothetical SOC(t) was first calculated for a wide range of hypothetical (OC/BC)_prim_ ratios (for ratios 0.1 to 15 in 0.1 steps). Then, the R-squared value (R^2^) between hypothetical SOC(t) and BC(t) was calculated for every hypothetical (OC/BC)_prim_ ratio, and the optimal (OC/BC)_prim_ ratio was chosen where the R^2^ was minimal. Because the air quality was strongly influenced by the plume from the wildfire during the event and by traffic emissions otherwise, two different (OC/BC)_prim_ ratios were used in our study. (OC/BC)_prim_ equal to 1.1 was determined as an optimal value for subperiods before and after the Camp Fire event in November 2018, and (OC/BC)_prim_ equal to 4.4 for the period strongly influenced by the wildfire (Figure A1).

The wavelength-dependent optical absorption was split between BC and brown carbon (BrC) using the two-component model [27,28,29,30]:

(5) $$b_{\mathrm{abs}}\left(\lambda ,\;t\right)=b_{\mathrm{abs}}^{\mathrm{BC}}\left(\lambda ,\;t\right)+b_{\mathrm{abs}}^{\mathrm{BrC}}\left(\lambda ,\;t\right)\;b_{\mathrm{abs}}^{\mathrm{BC}}\left(\lambda ,\;t\right)=b_{\mathrm{abs}}^{\mathrm{BC}}\left(\lambda_{0},t\right)\cdot {\left(\frac{\lambda }{\lambda_{0}}\right)}^{-{\mathrm{AAE}}_{\mathrm{BC}}}\;b_{\mathrm{abs}}^{\mathrm{BrC}}\left(\lambda ,\;t\right)=b_{\mathrm{abs}}^{\mathrm{BrC}}\left(\lambda_{0},t\right)\cdot {\left(\frac{\lambda }{\lambda_{0}}\right)}^{-{\mathrm{AAE}}_{\mathrm{BrC}}\left(t\right)}$$

We assumed the absorption Ångström exponent of BC (AAE_BC_) had a constant value of 1.15, as thoroughly discussed in a recent study from the Los Angeles basin [10]. Because AAE_BrC_(t) was allowed to vary with time, the two-component model (Equation (5)) did not have an analytical solution. To solve model (5), an assumption that BC was the only light-absorbing carbonaceous component at 880 nm was considered [10,28,29,31]:

(6) $$b_{\mathrm{abs}}\left(880,\;t\right)=b_{\mathrm{abs}}^{\mathrm{BC}}\left(880,\;t\right)\;b_{\mathrm{abs}}^{\mathrm{BrC}}\left(880,\;t\right)=0$$

Using assumption (6), the optical absorption by BrC at a specific wavelength can be calculated from model (5) as

(7) $$b_{\mathrm{abs}}^{\mathrm{BrC}}\left(\lambda ,\;t\right)=b_{\mathrm{abs}}\left(\lambda ,\;t\right)-b_{\mathrm{abs}}^{\mathrm{BC}}\left(\lambda ,\;t\right)=b_{\mathrm{abs}}\left(\lambda ,\;t\right)-b_{\mathrm{abs}}\left(880,\;t\right)\cdot {\left(\frac{\lambda }{880}\right)}^{-{\mathrm{AAE}}_{\mathrm{BC}}}$$

Spectral dependence of the absorption coefficient can be described by the bulk absorption Ångström exponent (AAE), which is calculated by fitting over all 7 wavelengths (AAE_7λ_). Besides the fraction of BrC absorption at 370 nm, the spectral dependence of $b_{\mathrm{abs}}^{\mathrm{BrC}}$ can be described by the AAE_BrC_, where $b_{\mathrm{abs}}^{\mathrm{BrC}}$ is fitted over 4 shortest wavelengths (370 nm to 590 nm).

### 2.3. Uncertainties

The detailed evaluation of uncertainties for TC–BC measurements and applied models was widely discussed in a recent paper [10]. Briefly, the uncertainty of TC concentrations measured by TCA08 was estimated to be up to 10%, while the uncertainty for BC concentration measured using AE33 was estimated to be 25%, where the largest part of uncertainty arose from the selected MAC_BC_ and C values. The uncertainty of the BC tracer model was also estimated to be 25%, and the BrC model yielded a discrepancy of 5% in the absorption coefficient of BrC at 370 nm.

### 2.4. Complementary Data

The smoke from Camp Fire extended over a broader area and affected air quality in Berkeley and a considerable fraction of northern California. To analyze the spatial extent of the Camp Fire on air quality in a broader area, the data from 4 publicly available databases were added:

1. Satellite images by NASA Worldview [32];
2. Back trajectories by the HYSPLIT model [33];
3. Smoke maps by NOAA Hazard Mapping System [34];
4. PM_2.5_ by Purple Air [35].

Appendix A contains daily images of spatial plume distribution in Northern California and calculated back trajectories from the Berkeley site between 7 and 21 November 2018 (Figure A2, Figure A3, Figure A4, Figure A5, Figure A6, Figure A7, Figure A8, Figure A9, Figure A10, Figure A11, Figure A12, Figure A13, Figure A14, Figure A15 and Figure A16). The back trajectories marking the path of air masses coming to the Berkeley site were calculated every 3 h by the HYSPLIT model for the last 72 h [33]. The GFS quarter-degree archive of meteorological data is used for the calculation.

Ground-based daily-averaged PM_2.5_ concentrations were estimated by Purple Air sensors (PASs). Owing to their low cost and, consequently, widespread use, PASs are useful for spatially visualizing the extent of pollution. Regardless of different corrections algorithms [36], the PAS biases could not be reduced entirely, and therefore, PASs were not used for mass closure in our study.

## 3. Results and Discussion

**Figure 2:**
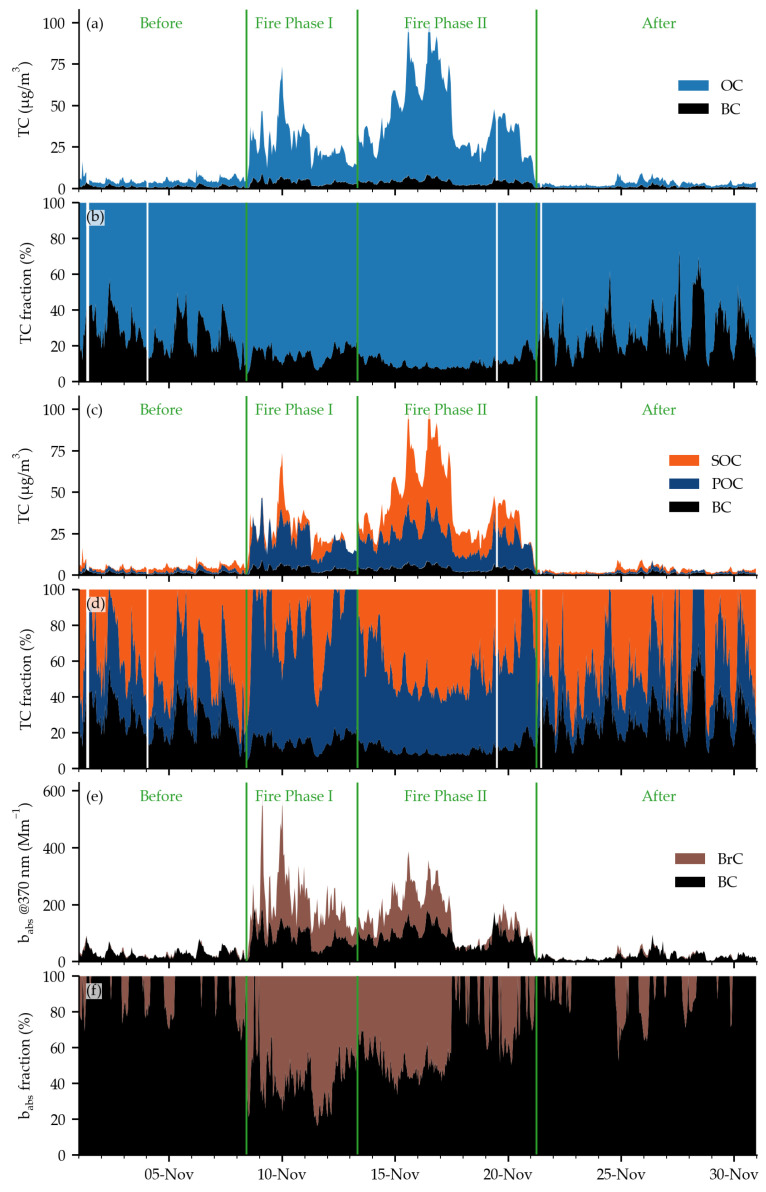
Time series of TC–BC measurements (**a**,**b**), CA apportionment (**c**,**d**), and absorption coefficient b_abs_ at 370 nm (**e**,**f**) in November 2018. Subplots (**a**,**c**,**e**) contain absolute values, and subplots (**b**,**d**,**f**) are presented in fractional contribution. White vertical lines are missing data, and green lines represent the split into phases: before the event, Fire Phase I, Fire Phase II, and after the event.

Camp Fire started on 8 November 2018, at around 6:15 a.m., approximately 145 km north of Sacramento, California. It was ignited by a faulty electric transmission line. The plume reached the Berkeley site in the next 4 h (Figure A4). The heavy rain on 21 November completely extinguished the fire (Figure A17) and washed out the pollution from the atmosphere. Figure 2 shows the time series of measured TC and BC (Figure 2a,b), apportionment of primary and secondary OC (Figure 2c,d), and apportionment of optical absorption to BC and BrC (Figure 2e,f).

**Figure 3:**
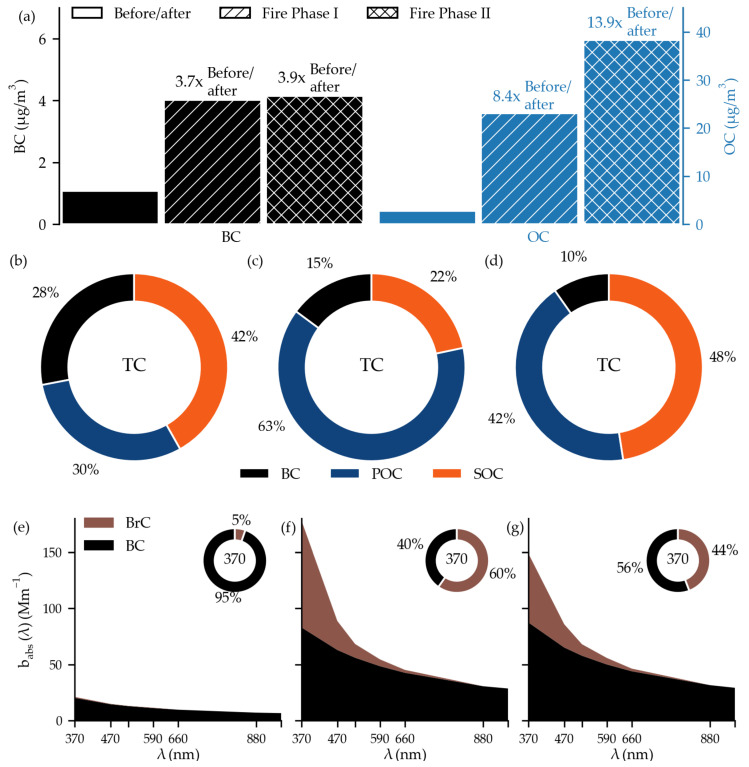
The upper row compares median BC and OC concentrations between different phases in November 2018 (**a**), where the bar height represents the median concentration and the numbers above the graph indicate the relative change. The middle row contains typical fingerprints for phases before and after Camp Fire event (**b**), during Fire Phase I (**c**), and during Fire Phase II (**d**). The median wavelength-dependent optical absorption is presented at the bottom for phases before and after Camp Fire event (**e**), during Fire Phase I (**f**), and during Fire Phase II (**g**). The pie charts in Figures (**e**–**g**) contain the average split of optical absorption between BC and BrC at 370 nm for the respective phases.

**Figure A2:**
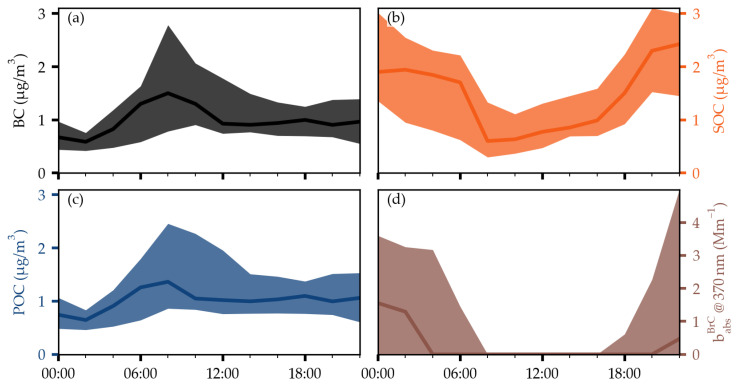
Diurnal profiles of BC (**a**), SOC (**b**), POC (**c**), and $b_{\mathrm{abs}}^{\mathrm{BrC}}$ (**d**) for typical November conditions in Berkeley before and after the Camp Fire event. The line represents the median value and the shaded area concentrations between the first and third quartiles.

Before and after the Camp Fire event in November 2018, it is possible to observe the typical daily cycle of CA in Berkeley (Figure 2 and diurnal profiles in Figure A2), comparable to the typical daily cycle from the Los Angeles basin [10]. BC represented approximately 40% of TC. The highest fraction of OC, up to 80% of TC, was observed during the nights (Figure 2b). During the day, primary CA (BC + POC) prevailed, and during the night, SOC contributed up to 70% of TC (Figure 2d). Before and after the Camp Fire event, the light absorption by BC strongly prevailed over BrC. BrC appeared only during the nights (Figure 2f) and, on average, represented approximately a 5% fraction of the light absorption at 370 nm in November 2018 (Figure 3e).

**Figure A3:**
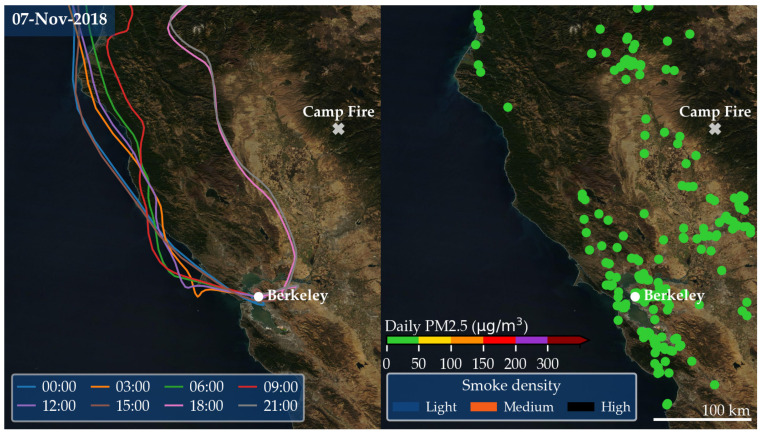
The spatial distribution of the smoke from the Camp Fire in Northern California on 7 November 2018. The satellite images by NASA Worldview [32] and back trajectories by the HYSPLIT model [33] are on the left, and Smoke maps by NOAA Hazard Mapping System [34] and daily averaged PM_2.5_ concentrations measured by Purple Air sensors [35] are on the right.

**Figure A4:**
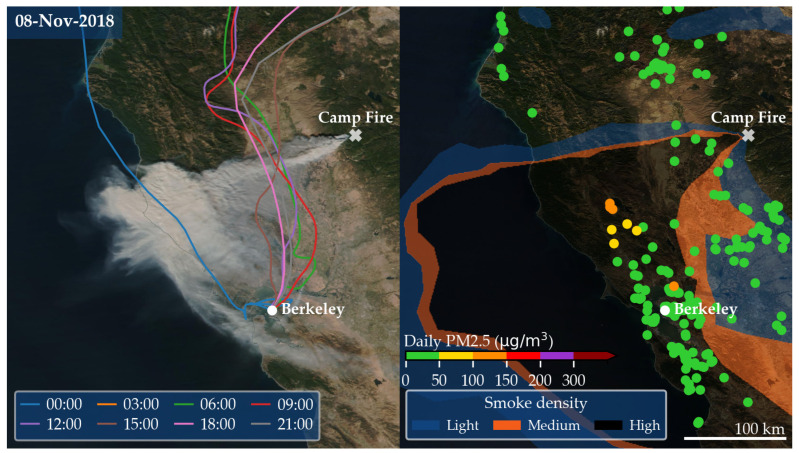
The spatial distribution of the smoke from the Camp Fire in Northern California on 8 November 2018. The satellite images by NASA Worldview [32] and back trajectories by the HYSPLIT model [33] are on the left, and Smoke maps by NOAA Hazard Mapping System [34] and daily averaged PM_2.5_ concentrations measured by Purple Air sensors [35] are on the right.

**Figure A5:**
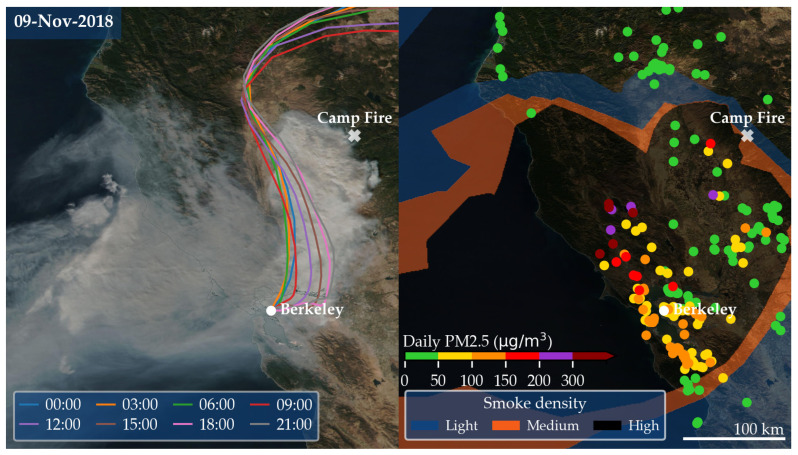
The spatial distribution of the smoke from the Camp Fire in Northern California on 9 November 2018. The satellite images by NASA Worldview [32] and back trajectories by the HYSPLIT model [33] are on the left, and Smoke maps by NOAA Hazard Mapping System [34] and daily averaged PM_2.5_ concentrations measured by Purple Air sensors [35] are on the right.

**Figure A6:**
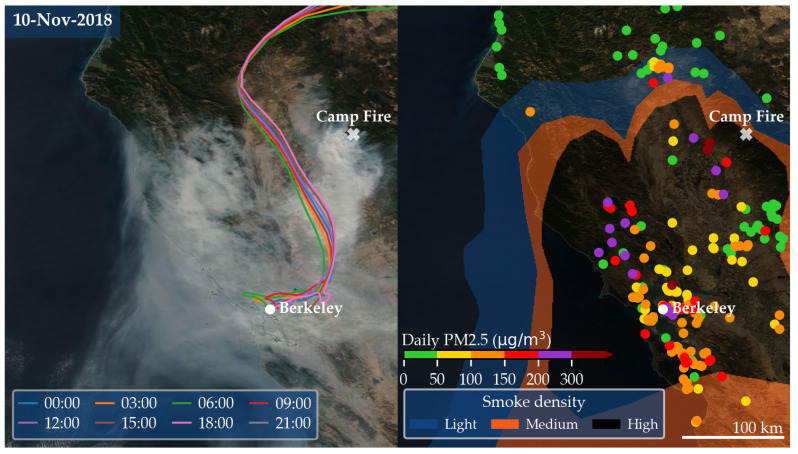
The spatial distribution of the smoke from the Camp Fire in Northern California on 10 November 2018. The satellite images by NASA Worldview [32] and back trajectories by the HYSPLIT model [33] are on the left, and Smoke maps by NOAA Hazard Mapping System [34] and daily averaged PM_2.5_ concentrations measured by Purple Air sensors [35] are on the right.

**Figure A7:**
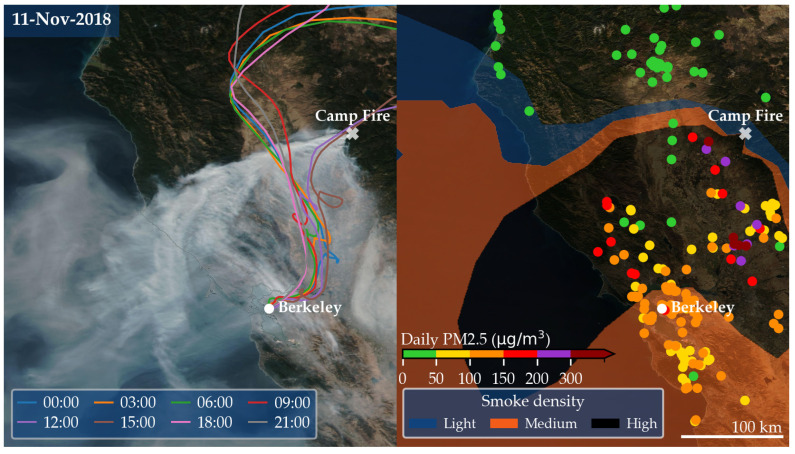
The spatial distribution of the smoke from the Camp Fire in Northern California on 11 November 2018. The satellite images by NASA Worldview [32] and back trajectories by the HYSPLIT model [33] are on the left, and Smoke maps by NOAA Hazard Mapping System [34] and daily averaged PM_2.5_ concentrations measured by Purple Air sensors [35] are on the right.

**Figure A8:**
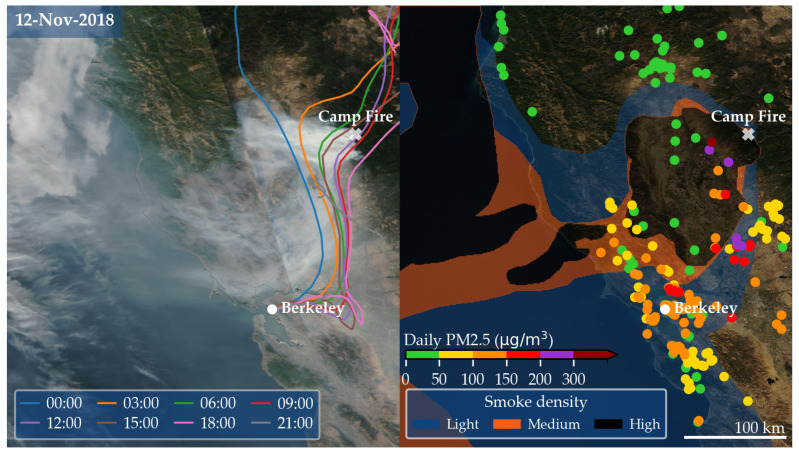
The spatial distribution of the smoke from the Camp Fire in Northern California on 12 November 2018. The satellite images by NASA Worldview [32] and back trajectories by the HYSPLIT model [33] are on the left, and Smoke maps by NOAA Hazard Mapping System [34] and daily averaged PM_2.5_ concentrations measured by Purple Air sensors [35] are on the right.

**Figure A9:**
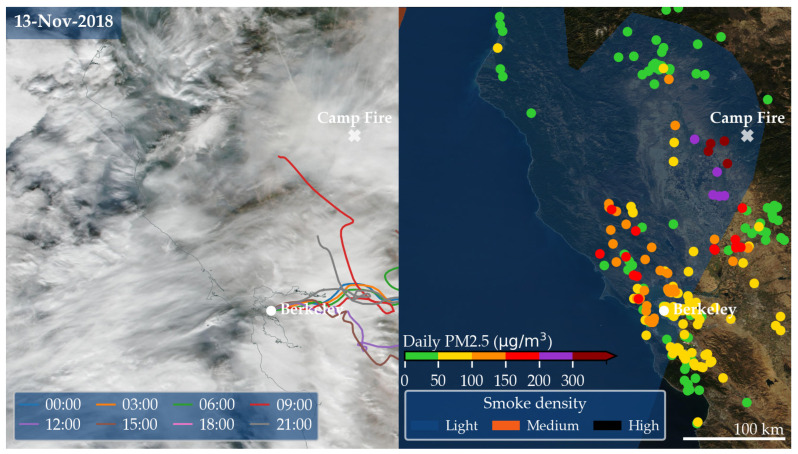
The spatial distribution of the smoke from the Camp Fire in Northern California on 13 November 2018. The satellite images by NASA Worldview [32] and back trajectories by the HYSPLIT model [33] are on the left, and Smoke maps by NOAA Hazard Mapping System [34] and daily averaged PM_2.5_ concentrations measured by Purple Air sensors [35] are on the right.

**Figure A10:**
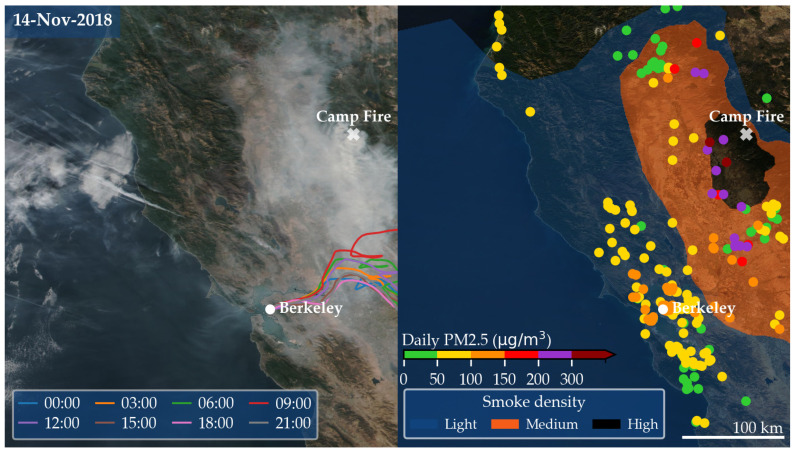
The spatial distribution of the smoke from the Camp Fire in Northern California on 14 November 2018. The satellite images by NASA Worldview [32] and back trajectories by the HYSPLIT model [33] are on the left, and Smoke maps by NOAA Hazard Mapping System [34] and daily averaged PM_2.5_ concentrations measured by Purple Air sensors [35] are on the right.

**Figure A11:**
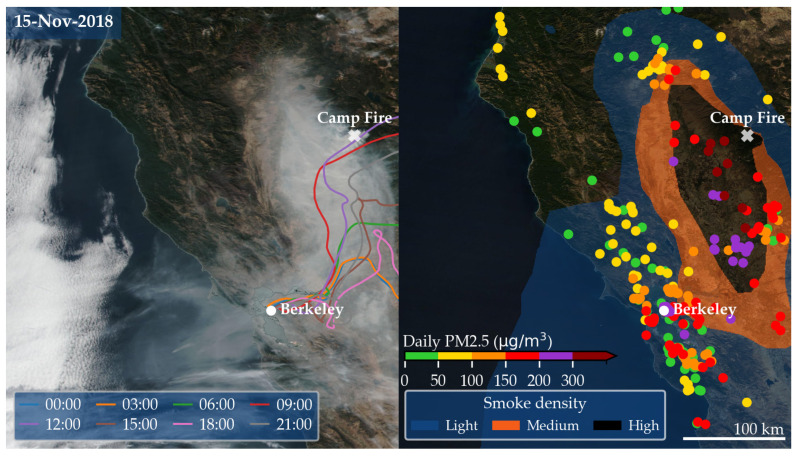
The spatial distribution of the smoke from the Camp Fire in Northern California on 15 November 2018. The satellite images by NASA Worldview [32] and back trajectories by the HYSPLIT model [33] are on the left, and Smoke maps by NOAA Hazard Mapping System [34] and daily averaged PM_2.5_ concentrations measured by Purple Air sensors [35] are on the right.

**Figure A12:**
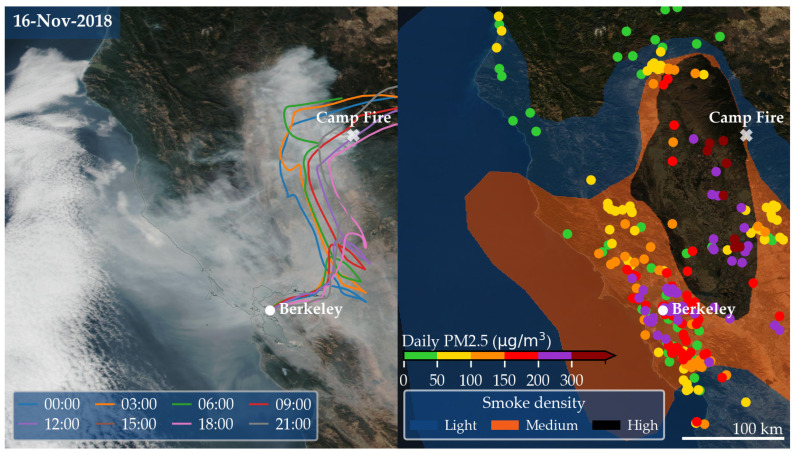
The spatial distribution of the smoke from the Camp Fire in Northern California on 16 November 2018. The satellite images by NASA Worldview [32] and back trajectories by the HYSPLIT model [33] are on the left, and Smoke maps by NOAA Hazard Mapping System [34] and daily averaged PM_2.5_ concentrations measured by Purple Air sensors [35] are on the right.

**Figure A13:**
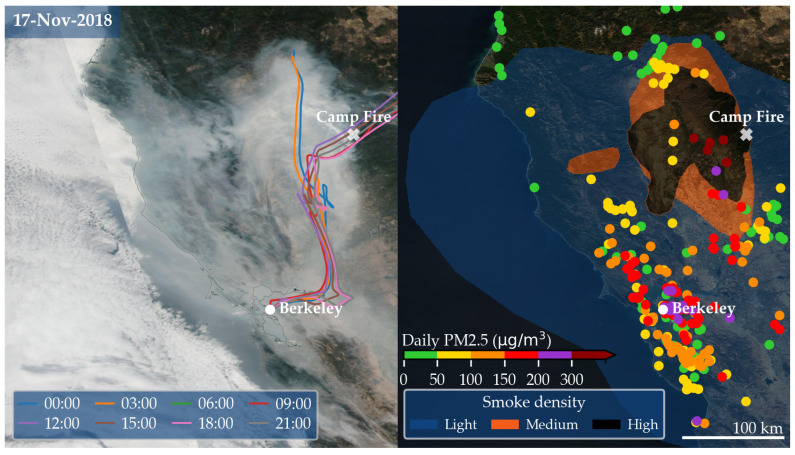
The spatial distribution of the smoke from the Camp Fire in Northern California on 17 November 2018. The satellite images by NASA Worldview [32] and back trajectories by the HYSPLIT model [33] are on the left, and Smoke maps by NOAA Hazard Mapping System [34] and daily averaged PM_2.5_ concentrations measured by Purple Air sensors [35] are on the right.

**Figure A14:**
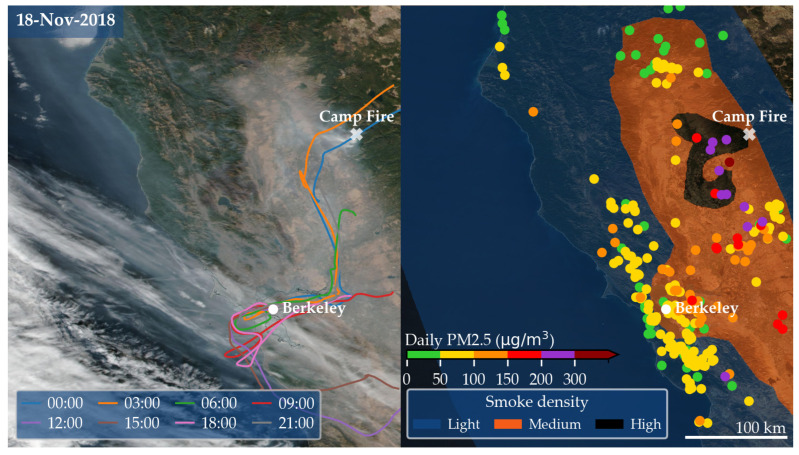
The spatial distribution of the smoke from the Camp Fire in Northern California on 18 November 2018. The satellite images by NASA Worldview [32] and back trajectories by the HYSPLIT model [33] are on the left, and Smoke maps by NOAA Hazard Mapping System [34] and daily averaged PM_2.5_ concentrations measured by Purple Air sensors [35] are on the right.

**Figure A15:**
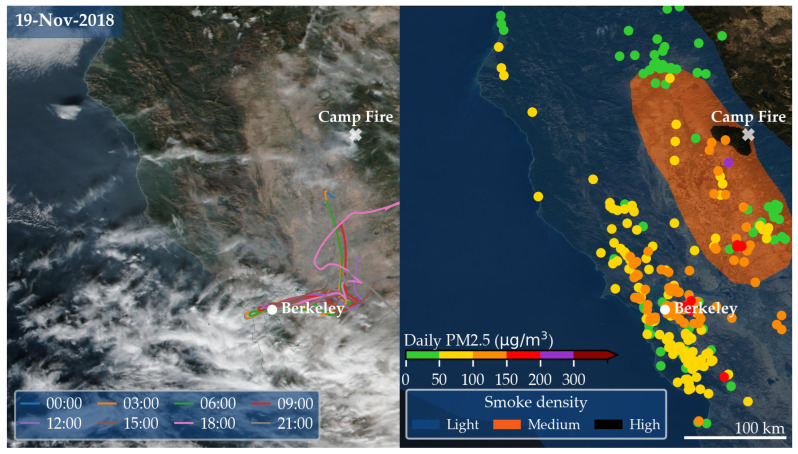
The spatial distribution of the smoke from the Camp Fire in Northern California on 19 November 2018. The satellite images by NASA Worldview [32] and back trajectories by the HYSPLIT model [33] are on the left, and Smoke maps by NOAA Hazard Mapping System [34] and daily averaged PM_2.5_ concentrations measured by Purple Air sensors [35] are on the right.

**Figure A16:**
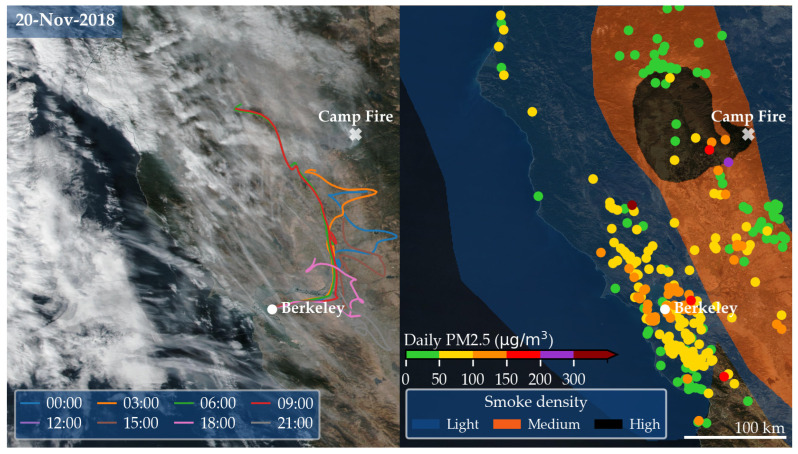
The spatial distribution of the smoke from the Camp Fire in Northern California on 20 November 2018. The satellite images by NASA Worldview [32] and back trajectories by the HYSPLIT model [33] are on the left, and Smoke maps by NOAA Hazard Mapping System [34] and daily averaged PM_2.5_ concentrations measured by Purple Air sensors [35] are on the right.

**Figure A17:**
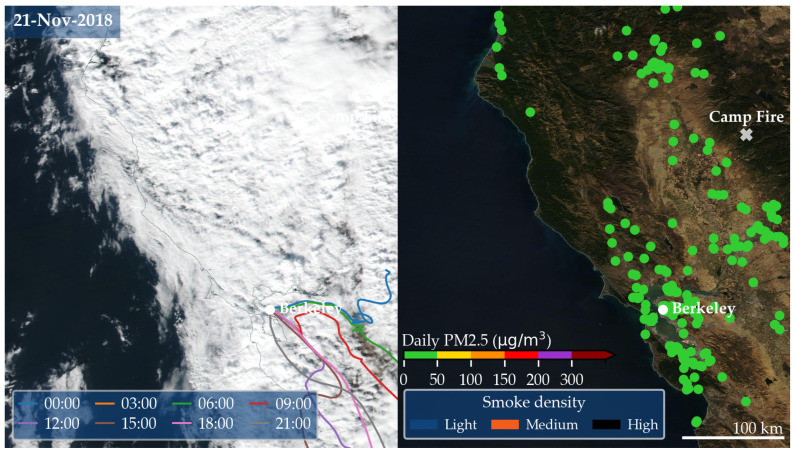
The spatial distribution of the smoke from the Camp Fire in Northern California on 21 November 2018. The satellite images by NASA Worldview [32] and back trajectories by the HYSPLIT model [33] are on the left, and Smoke maps by NOAA Hazard Mapping System [34] and daily averaged PM_2.5_ concentrations measured by Purple Air sensors [35] are on the right.

The air quality significantly deteriorated when the wildfire plume reached Berkeley (Figure 2a). We split this period into two phases: Fire Phase I and Fire Phase II (denoted with vertical green lines in Figure 2). In Fire Phase I, the fire was active due to high vegetation fuel loading, and this period can be characterized by the flaming phase of the fire. The high plume rise and strong winds allowed the plume to be transported over long distances (Figure A4, Figure A5, Figure A6, Figure A7, Figure A8 and Figure A9). The smoldering and spot fires were more typical for Fire Phase II. The initial plume was already dispersed, while the intake of fresh smoke was lower for more distanced places such as the Berkeley site (Figure A10, Figure A11, Figure A12, Figure A13, Figure A14, Figure A15 and Figure A16). The air masses were coming to the Berkeley site from the northeast direction most of the time when the fire was active (back trajectories in Figure A4, Figure A5, Figure A6, Figure A7, Figure A8, Figure A9, Figure A10, Figure A11, Figure A12, Figure A13, Figure A14, Figure A15 and Figure A16). The daily averaged PM_2.5_ concentrations, measured by Purple Air sensors, exceeded 200 µg/m^3^ in Berkeley and 300 µg/m^3^ in places nearer the fire (Figure A3, Figure A4, Figure A5, Figure A6, Figure A7, Figure A8, Figure A9, Figure A10, Figure A11, Figure A12, Figure A13, Figure A14, Figure A15, Figure A16 and Figure A17).

In Fire Phase I, the median TC concentrations increased from 3.5 µg/m^3^ to 23.9 µg/m^3^ with a maximum hourly and 24 h average value of 73.8 µg/m^3^ and 37.1 µg/m^3^, respectively (Figure 2a). In Fire Phase II, even higher TC concentrations were observed: 37.0 µg/m^3^, 102.9 µg/m^3^, and 75.0 µg/m^3^ for the median, hourly maximum, and maximum 24 h average TC, respectively. During both fire phases, the 24 h average TC exceeded the WHO-recommended PM_2.5_ daily limit of 15 µg/m^3^ [37] every day. In 7 of these 13 days, the 24 h average TC concentrations were two times higher than the limit. It is worth noting that the TC concentration only represents the carbon content in carbonaceous aerosols. In addition, the PM_2.5_ may be even higher due to other non-carbonaceous particles in the aged wildfire plume. While the fractional increase in median BC concentrations against the median BC value before and after the event was comparable in both fire phases (3.7 times in Fire Phase I and 3.9 times in Fire Phase II—Figure 3a), the pattern of OC increase was different. Compared to periods before and after the Camp Fire event, median OC increased 8.4 and 13.9 times in Fire Phases I and II, respectively (Figure 3a). This agrees with the study of Zhang et al. [7], where they reported more than 10 times higher OA concentration during the period affected by the wildfire than the background. Increase in the OC fraction in Fire Phase II infers more OC was formed secondarily. We confirmed this with the BC tracer model: during Fire Phase I, 63% of TC was POC (Figure 3c). Similarly, Palm et al. [19] reported the dominant POA role during the fire’s starting phase. In Fire Phase II, the most considerable fraction of TC was SOC (48%) (Figure 3d).

The fraction of the light absorbed by BrC increased significantly during the fire period compared to the typical situation before and after the event (Figure 2e,f). The maximum fraction of light absorbed by BrC at 370 nm was observed on 11 November when it reached 80% (Figure 2f). The median BC absorption coefficient at 370 nm was comparable between both fire phases—82.9 Mm^−1^ and 87.4 Mm^−1^ in Fire Phases I and II, respectively. The median BrC absorption coefficient at 370 nm decreased from 94.6 Mm^−1^ to 61.2 Mm^−1^ in Fire Phase II (Figure 3f,g). This resulted in a lower fraction of light absorbed by BrC at 370 nm—it decreased from 60% in Fire Phase I to 44% in Fire Phase II (Figure 3f,g). The decrease in BrC absorption is most probably connected to the photobleaching of BrC. The net decrease in BrC absorption with aging due to photobleaching is also consistent with other studies [15,17,18,38,39]. The lowest fraction of light absorbed by BrC was found between 17 and 19 November, with less than a 20% share (Figure 2f). The lower smoke intensity can also be seen in satellite images (Figure A14 and Figure A15).

**Figure 4:**
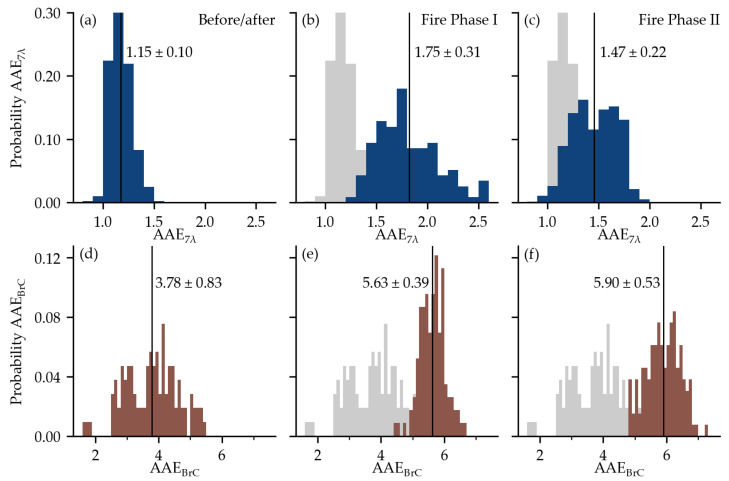
The probability distribution of AAE_7λ_ in blue (**a**–**c**) and AAE_BrC_ in brown (**d**–**f**). Subplots (**a**,**d**) contain the distributions before/after the Camp Fire event, (**b**,**e**) for Fire Phase I, and (**c**,**f**) for Fire Phase II. The gray, shadowed distributions in (**b**,**c**,**e**,**f**), are added for orientation and represent the typical distributions before/after the Camp Fire event from (**a**,**d**). The vertical black lines represent mean values; the value of the standard deviation for each distribution is also added to the plots.

Figure 4 contains analyses of the AAE for bulk absorption (AAE_7λ_) and BrC (AAE_BrC_). The mean AAE_7λ_ was equal to 1.15 before and after the air quality was affected by Camp Fire smoke (Figure 4a), and the mean value of AAE_BrC_ for the same periods was equal to 3.78 (Figure 4d). Both AAE_7λ_ and AAE_BrC_ increased when the air was affected by Camp Fire smoke (Figure 4b–c,e–f). The AAE_BrC_ was slightly (statistically significant) higher in Fire Phase II compared to Fire Phase I—the mean AAE_BrC_ increased from 5.63 to 5.90 with a *p*-value of 0.01 for Mood’s median test (Figure 4e,f). The opposite pattern was observed for AAE_7λ_—it decreased in Fire Phase II (Figure 4b,c) due to the higher fraction of light absorption by BC in Fire Phase II (Figure 3f,g). The decrease in AAE_7λ_ during the later wildfire stage is consistent with other wildfire studies of aged plumes in Northern America [38,39].

## 4. Conclusions

Wildfires are an uncontrollable source of carbonaceous aerosols. Wildfire smoke can cause high levels of PM_2.5_ in urban areas, exceeding the WHO guidelines for several days. Exposure to such high levels of PM_2.5_ can adversely affect respiratory health, especially for vulnerable groups such as children, the elderly, and people with chronic respiratory diseases. Different, non-typical measures must be applied to reduce the health risks of the population, such as closing schools and other public objects. People are usually advised to spend most of their time indoors and reduce the natural ventilation of the buildings they occupy. Real-time and highly time-resolved measurements are required to make appropriate decisions about the measures and risks.

In our study, the CASS, a combined unit of a TCA and an Aethalometer, was used to measure the influence of the smoke from the distant Camp Fire on local air quality in Berkeley. High-time-resolution measurements, in combination with numerical models such as the BC tracer and BrC model, allow us to apportion the pollution in real-time. The 24 h average TC concentration exceeded the recommended WHO PM_2.5_ limit every day when the Berkeley site was affected by the wildfire smoke from Camp Fire, and the 24 h average concentrations were two times higher than the limit in 7 of these 13 days. Median BC concentrations increased approximately four times above the pollution level typical for Berkeley. The median OC increased by 8.4 in the first, more active fire phase when flaming is expected to dominate. Later, when more smoldering is expected, the median OC increase was even higher, 13.9 times above the background concentrations, suggesting a more intense formation of SOA during the later fire phase.

The results of apportionment models that can be run in real-time can be an essential input for short-term air quality prediction models. On the other hand, we showed an increased contribution of BrC to total light absorption at short wavelengths during the event. Therefore, the fraction of the absorbed light by BrC can be used as a marker for detecting the influence of wildfire plumes. While the fractional contribution of BrC to light absorption before and after the wildfire event was low (approximately 5%), it increased up to 50% during the event. Considering that the magnitude and variability of BrC absorption are still uncertain and poorly represented in climate models, we believe the multiwavelength measurements of aerosol optical properties can help improve the parameterization of BrC absorption in climate models.

## Data Availability

Not applicable.

## Appendix A

Figure A1 contains the results of the BC tracer model. The minimum R-squared method is used to estimate the optimal (OC/BC)_prim_ ratio [25,26].

A significant shift to higher OC/BC ratios can be observed when the air quality was strongly influenced by the smoke from Camp Fire in Figure A1. Before and after the Camp Fire event (Figure A1a), 75% of all OC/BC ratios were lower than 4.1, with a median value of 2.9, and 75% of all OC/BC ratios were higher than 4.4 during the Camp Fire event (Figure A1b), with a median value of 5.5. Lower OC/BC (OC/EC) ratios are typical for traffic sites. For example, Zhou et al. [40] collected typical OC/EC ratios for tunnel experiments between 0.3 and 1.6. Higher OC/EC ratios from biomass burning are expected—for example, in work by Tian et al. [41], the OC/EC ratios from different biomass-burning experiments were between 2.4 and 9.1.

Figure A2 contains typical diurnal profiles of BC, SOC, POC, and $b_{\mathrm{abs}}^{\mathrm{BrC}}$, averaged for periods before and after the Camp Fire event.

Figure A3, Figure A4, Figure A5, Figure A6, Figure A7, Figure A8, Figure A9, Figure A10, Figure A11, Figure A12, Figure A13, Figure A14, Figure A15, Figure A16 and Figure A17 contain the spatial distribution of the smoke from the Camp Fire in Northern California. The data from four publicly available datasets are joined in these figures: Satellite images by NASA Worldview [32] and back trajectories by the HYSPLIT model [33] on the left, and Smoke maps by NOAA Hazard Mapping System [34] and daily averaged PM_2.5_ concentrations measured by Purple Air sensors [35] on the right.
